# The diagnosis of acute intermittent porphyria combined with seizures: Case report

**DOI:** 10.1097/MD.0000000000036167

**Published:** 2023-12-15

**Authors:** Zhen Wang, Liniu Cheng, Xianyang Liang, Han Jiang, Ruile Shen

**Affiliations:** a Department of Neurology, The First Affiliated Hospital, and College of Clinical Medicine of Henan University of Science and Technology, Luoyang, Henan, China.

**Keywords:** abdominal pain, case report, neuropsychiatric, porphyria, urinary porphyrin

## Abstract

**Rationale:**

Acute intermittent porphyria (AIP) is a rare metabolic disorder affecting heme production due to enzyme porphobilinogen deaminase deficiency. Diagnosing acute intermittent porphyria is difficult because its symptoms interrelate with those of other common diseases. When AIP is combined with seizures, the diagnosis process is more complicated. This case report shows all tests and criteria used to arrive at the final stage of diagnosis.

**Patient concerns:**

The patient complained of severe abdominal pain, nausea, vomiting, and intermittent convulsions. Her medical history shows she had abdominal pain, mainly dull pain in the left upper abdomen.

**Diagnoses:**

Different symptomatic tests were done, and the cause of her symptoms was uncertain. A urine sun drying test was then done and confirmed the presence of porphyrin used to diagnose AIP. A genetic test was done after the patient was discharged, and AIP diagnosis was confirmed.

**Interventions:**

Acute intermittent porphyria treatment was administered.

**Outcomes:**

The patent recovered fully.

**Lessons:**

It is essential to consider acute intermittent porphyria diagnosis in patients having unexplained severe abdominal pain associated with neurological and psychiatric symptoms. Since AIP is a rare disease with a high mortality rate when not treated early, Clinical practices should include AIP as one of the tests done on patients showing these symptoms at an early stage. The fastest way to identify this is to conduct a urine test. The change of color from brown to reddish color is a diagnostic indicator of AIP. This strategy helps reduce misdiagnoses and delayed treatment of the right disease.

**Patient’s perspective:**

The patient was pleased that her extreme pain was over now that she had received the proper medication for her long-time illness. She was happy to have stopped being under various medications she had been on for a long time, and the issue recurring.

## 1. Introduction

Acute intermittent porphyria (AIP) is the most common acute porphyria hereditary disorder characterized by aberrations in the biosynthesis of heme.^[1]^ It is a critical molecule in various biological processes. AIP is an autosomal dominant genetic anomaly from enzyme deficiencies such as hepatic delta-aminolevulinic acid dehydratase and porphobilinogen deaminase. These enzymes orchestrate the intricate orchestration of porphyrin metabolism.^[2]^ This intricate enzymatic deficit accumulates porphyrin precursors, engendering physiological consequences. Clinical manifestations of AIP are diverse, ranging from gastrointestinal symptoms and neurological manifestations to skin photosensitivity.^[3]^ The hallmark of AIP lies in its propensity to induce recurrent abdominal pain accompanied by a constellation of neurological and psychiatric symptoms.^[4]^ Notably affecting individuals between the ages of 20 and 40, AIP’s clinical presentation can often mirror various other conditions, rendering its diagnosis particularly challenging.

Moreover, the potential overlap of symptoms with other medical entities may lead to misdiagnosis, thereby delaying appropriate management and treatment.^[5,6]^ Porphyria is caused by an enzyme defect in the heme biosynthetic pathway and is diagnosed primarily by specific biochemical patterns of porphyrins and their precursors in Urine, feces, and blood.^[1]^ Clinical manifestations in porphyria patients are associated with cumulative patterns of porphyrins, precursors, and derivatives, including abdominal, neurological, psychiatric, endocrine, cardiovascular symptoms, liver damage, and skin sensitivity to light.^[7,8]^ The major types of Porphyrias are X-linked sideroblastic anemia, ALA dehydratase deficiency porphyria (ADP), acute intermittent porphyria (AIP), congenital erythropoietic porphyria, delayed cutaneous porphyria, hereditary porphyria (HCP), mixed porphyria (VP) and lastly erythropoietin protoporphyria (EPP). These types can be divided according to the kind of pathogenesis, which can either be acute or non-acute type.^[9]^ In symptomatic acute hepatic porphyria, there is an increased synthesis of porphyrin precursors and porphyrins, previously considered neurotoxic. In the non-acute type, accumulated Porphyrins can cause skin sensitivity to light (photodermatoses) and severe liver damage. Porphyria has an early onset and is clinically rare, making its diagnosis challenging.^[10]^ This report shows a case of acute intermittent porphyria, which was diagnosed and treated in our hospital.

## 2. Patient information

The patient was a 20-year-old Chinese woman who worked as a housekeeper. The patient complained of severe abdominal pain, nausea, vomiting, and intermittent convulsions. From her family medical history, there was no extraordinary condition. There was no history of encephalitis, head trauma, or hepatitis tuberculosis, and no history of febrile seizure. The patient was in good health, with full-term spontaneous delivery. On medical history, the patient had had abdominal pain in the past three years, mainly dull pain in the left upper abdomen. It was occasionally accompanied by nausea, vomiting, poor feeding, no diarrhea, and fever. Each attack lasted from several hours to several even days. In her consultation with different hospitals, gastritis was diagnosed, and she received oral gastritis drug treatment. However, the effect was not good as the patient developed severe facial rashes at 17. Some of which were large and ruptured. She was treated for “acne” in the dermatology department.

## 3. Clinical findings

From the physical examination, the patient’s vomit was stomach contents, and she had no diarrhea, hematemesis, fever, shoulder and back radiating pain, chest tightness, chest pain, or dyspnea.

Admission examination was as follows: T37.2°C P 130 times/min R 25 times/min BP 140/100 mm Hg Clear, poor energy, no obvious dry or wet rales in the lungs, cardiac auscultation of the rhythm, no pathological murmurs in the valve areas, soft abdominal muscles, evident abdominal tenderness throughout the abdomen, suspected positive rebound pain, negative Murphy sign, negative percussion pain in the liver area, negative percussion pain in the renal area, suspected positive for Dai Mai’s point tenderness but there was no edema in both lower extremities. Blood gas was tested in our hospital on the same day. It showed that the partial pressure of blood carbon dioxide and blood oxygen was 32.00 and 139.00 mm Hg, respectively, the standard value of bicarbonate was 21.70 mmol/L, the actual value of alkali remaining was −5.00 mmol/Sodium ion was 125.00 mmol/L, and potassium ion was 4.40 mmol/L. A blood routine test on the same day showed that the number of white blood cells was 11.65 × 10^9/L, Neutrophils 10.95 × 10^9/L, and lymphocytes 0.61 × 10^9/L. The percentage of neutrophils was 94.00%, hemoglobin 107.00g/L, and hematocrit 33.00%. Blood Biochemistry tested in our hospital on the same day showed that amylase 211 U/L, sodium 129.7 mmol/L, chlorine 94.8 mmol/L, aspartate aminotransferase 45 U/L, Oxo butyrate dehydrogenase 186 U/L, and phosphocreatine kinase 551 U/L.

## 4. Timeline

The patients visited the hospital on February 23, 2021, and underwent several tests, as shown in Table 1, before the diagnosis was established on March 4, 2021. Throughout the period, she received symptomatic treatments to curb the abdominal pain and control the seizures, and the patient was then discharged from the hospital.

**Table 1:** Diagnoses and intervention milestones.

Historical dates	Diagnoses	Interventions
February 23, 2021	Chest & Renal CT done showed dense bilateral renal pelvis	The patient admitted to the emergency department and Fluid rehydration and symptomatic support treatment administered
February 24, 2021	Sudden limb convulsions, closed teeth, binocular gaze, and repeated seizures	Oral diazepam application administered.
February 27, 2021	Seizures reoccurred. Head CT was normal. Head MRI showed abnormalities	Patient transferred to neurology intensive care unit
March 4, 2021	The symptoms reoccurred Abdominal CT showed possible of incomplete intestinal obstruction	Enema and antispasmodic treatment administered.
March 9, 2021	Urine analysis done	Addition of Delixin to regulate gastrointestinal nerves, gabapentin, continuous intravenous drips of hyoscyamine, and lidocaine for pain relief. Patient discharged from the hospital
April 1, 2021	Genetic test results after discharge	Genetic test done and AIP was diagnosed. Patient was contacted and informed. Heme therapy prescribed to the patient.
April 15, 2021	Follow up check up	Patient did not have an attack for 2 weeks. Patient advised to visit the hospital in case of any attack and ensure she shows the diagnosis.

AIP = acute intermittent porphyria, CT = computerized tomography.

## 5. Diagnostic assessment

**Figure 1.**
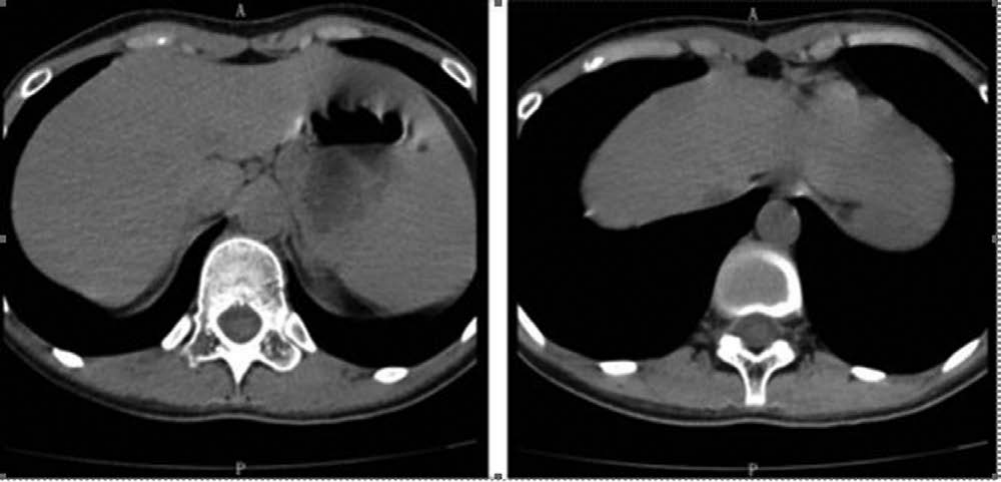
Bilateral adrenal CT scan with kidney findings showing probabilities of papillae caused by calcium milk deposition. adrenal shows no abnormalities; however, a significant amount of calcium milk deposit affects both kidneys. CT = computerized tomography.

**Figure 2.**
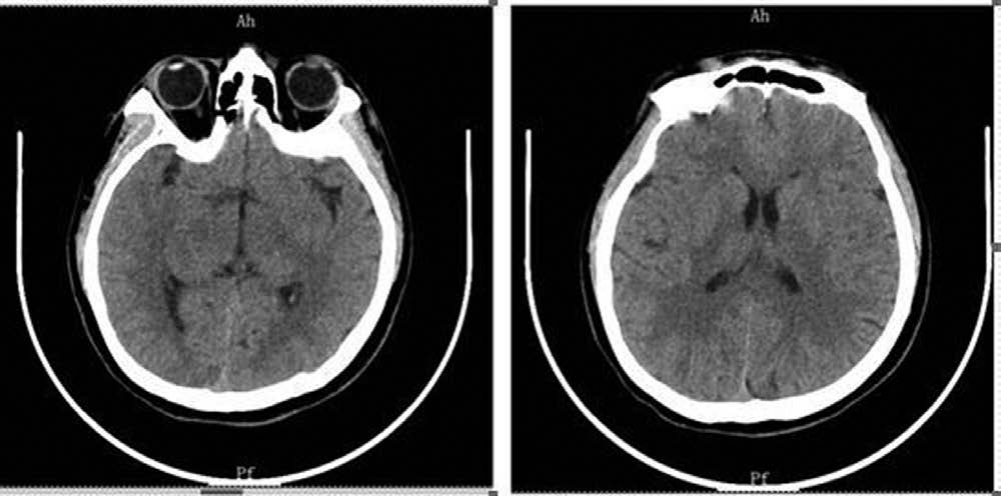
Head CT scan shows no abnormalities and seizures. The figure shows no identity of causes of seizure nor has any abnormalities. CT = computerized tomography.

The patient underwent chest computerized tomography (CT), hepatobiliary, pancreatic, spleen, and renal ultrasound at the local county hospital, and no abnormalities were found. Renal CT showed that the bilateral renal pelvis was dense, as shown in Figure 1, considering calcium salt deposition. On February 24, 2021, at 00:07, the patient had sudden limb convulsions, closed teeth, binocular gaze, and repeated seizures. Head CT showed no apparent abnormalities, as shown in Figure 2. On February 27, an invited neurologist advised that to control the seizures, the patient be transferred to the neurology intensive care unit for close monitoring.

**Figure 3.**
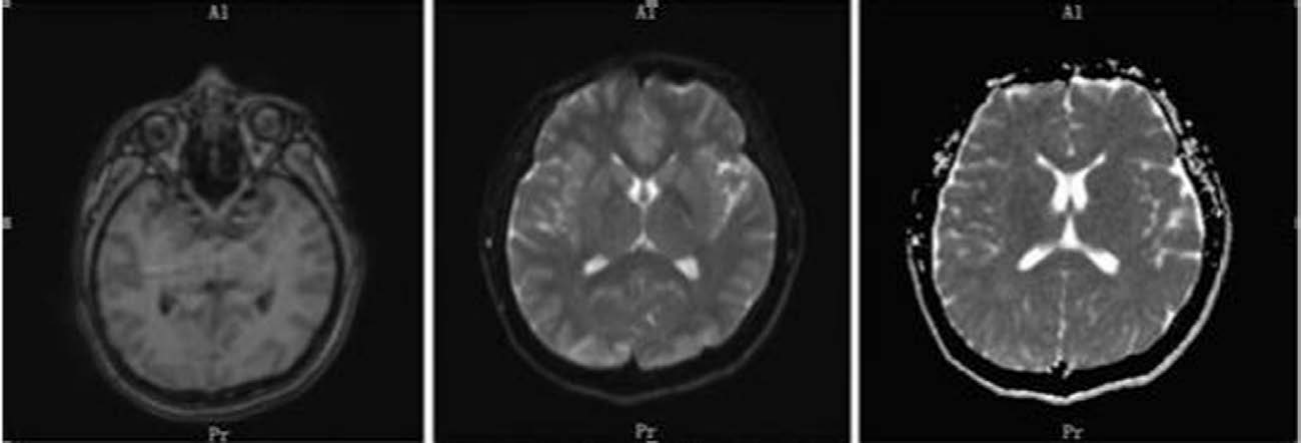
MRI scan of head revealing frontal and occipital lobe abnormalities with suspected posterior reversible encephalopathy syndrome characterized by reversible brain edema. MRI = magnetic resonance imaging.

**Figure 4.**
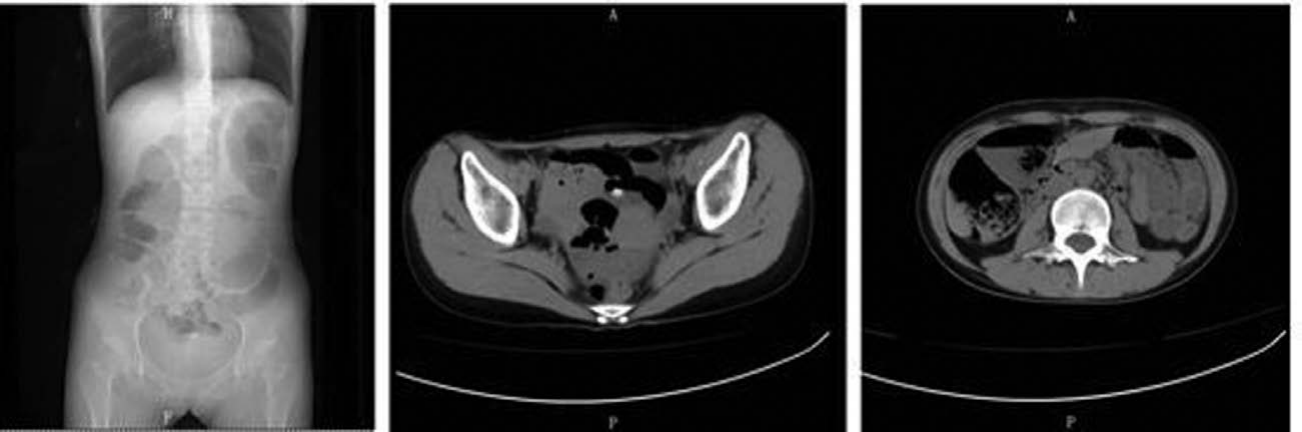
Abdominal CT scan findings: figure shows incomplete intestinal obstruction vs acute pancreatitis exclusion. CT = computerized tomography.

**Figure 5.**
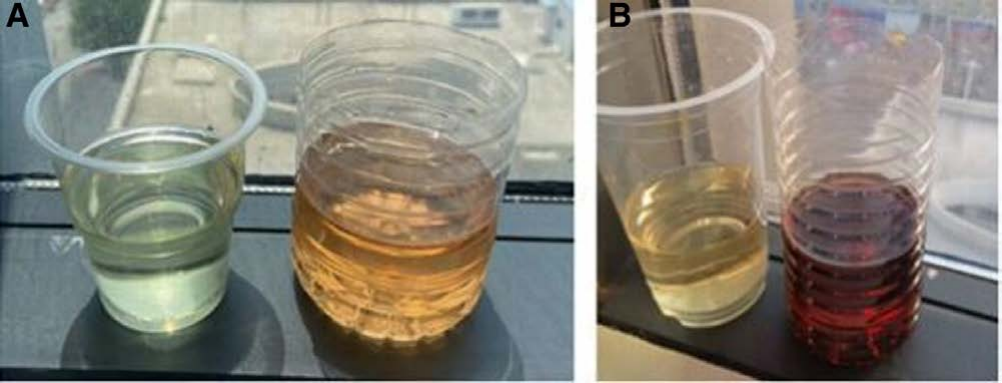
Comparison of urine colour before and after drying in the diagnosis of acute intermittent porphyria. The results show a comparison between the patient’s Urine (right) and the patient’s mother’s (left); visual comparison shows how the color of the patient’s Urine changed color, depicting the clinical manifestation of Acute Intermittent porphyria.

**Figure 6.**
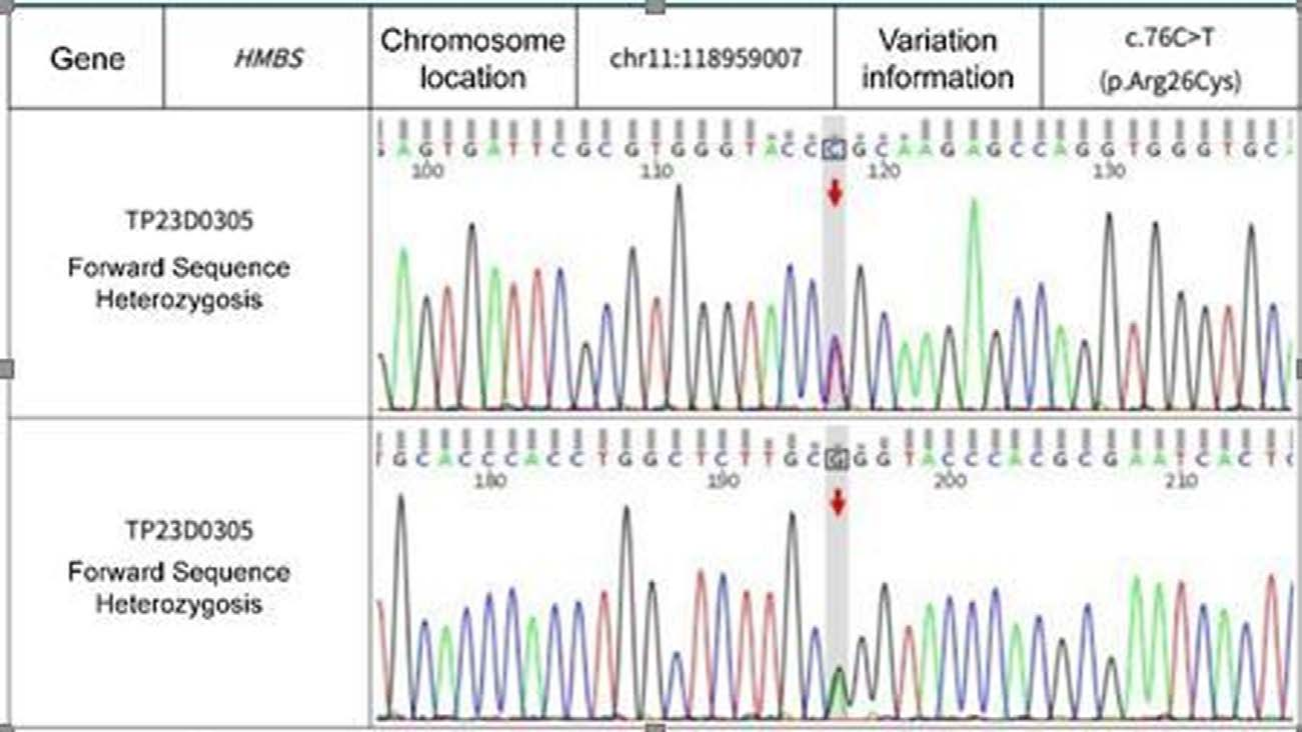
Genetic test results for suspected pathogen variant in the diagnosis of AIP. The results confirm the presence of missense mutation, a variant associated with AIP, confirming the diagnosis of AIP. AIP = acute intermittent porphyria.

Repeat head magnetic resonance imaging showed multiple abnormal signals. There were shadows in the white matter junction area of the frontal lobe and parietal occipital lobe on both sides, inflammation, and posterior reversible encephalopathy syndrome, as shown in Figure 3. The lumbar puncture CSF test is not exceptional, and autoimmune encephalitis-related antibodies are negative. On March 4, the patient was transferred to the second ward of the Neurology department. She intermittently had abdominal pain, mainly periumbilical pain, accompanied by nausea, vomiting, and loss of appetite. A reexamination of amylase came out normal. After the gastrointestinal surgery consultation, an Abdominal CT was done and showed the possibility of incomplete intestinal obstruction, as shown in Figure 4. Enema and antispasmodic treatment were administered, and the patient experienced reacted negatively. She experienced abdominal pain intermittently for several hours that night. After consultation with relevant departments, the basis for acute pancreatitis was insufficient as there was incomplete intestinal obstruction. Due to the limitations of the incomplete intestinal obstruction condition, it was impossible to complete further the measurement of Urine PBG, urine porphyrin, and hematoporphyrin. The symptoms did not improve significantly after enema and spasmolytic treatment. Abdominal ultrasound and gastroscopy did not show any abnormalities. The urine test analysis was done on 09-03-2021. A urine test sample was taken from the patient and her mother. Both samples were left to dry under sunlight. The patient’s sample was brown before drying, and after drying, it changed to a reddish color, as shown in Figure 5 A and B. The result was a positive indicator to consider the possibility of acute intermittent porphyria. The reddish/purple coloration of Urine happens because of porphyrin presence combined with their precursors, most often porphobilinogen (PBG) in Urine. These compounds oxidize when subjected to sunlight and cause Urine to change color. After being discharged and Genetic test results taken on 2021.04.01 after discharge, as shown in Figure 6, shows the presence of HMBS chr11:118959007 Exon2 NM_000190.4:c.76C > T (p.Arg26Cys), which is a missense mutation expected to turn the 26th amino acid Arg of the encoded protein into Cys. Multiple literature reports have detected this variant in various patients with acute intermittent haemo porphyria, and functional studies have shown that this variant decreases the encoded protein’s activity to about 5%. Multiple literature reports have reported the detection of variant P in numerous patients with acute intermittent haemo porphyria. Arg26His and p.Arg26Leu; ESP6500siv2_ALL, Thousand Human Genome (1000g2015aug_ALL) and dbSNP147 databases are omitted; Bioinformatics software predicts that it is more likely to cause disease. The variant is considered to be a suspected pathogenic variant. Based on the above evidence, the patient’s diagnosis of acute intermittent porphyria is precise.

## 6. Therapeutic intervention

Due to the dense renal pelvis from the CT scan, fluid rehydration and symptomatic support treatment were administered on February 23, 2021 at 16:00. However, abdominal pain did not subside. During the infusion process in the prefecture hospital, the patient had convulsions accompanied by closed teeth, eyes staring up, lost consciousness, no incontinence, and was foaming at the mouth. The convulsion lasted 2–3 minutes, and then she recovered. The convulsions reappeared half an hour later with the same symptoms and duration. The patient was admitted to the emergency department of our hospital. Following the repeated seizures and sudden limb convulsions on February 24, 2021 the patient was given a diazepam application, and the seizures were contained but later reoccurred.

On February 27, 2021 at the neurology intensive care unit, the patient received anti-epileptic treatment, consciousness was fully regained, and no more seizures occurred to the patient. On March 4, after the abdominal CT showed incomplete intestinal obstruction, the enema and antispasmodic treatment were administered, and the patient reacted negatively. She experienced abdominal pain intermittently for several hours that night. The symptoms did not improve significantly after enema and spasmolytic treatment. A large amount of intravenous sugar and arginine supplementation was given for the management of the attacks and to help the patient regain energy. Levetiracetam was administered to control seizures, and further genetic testing was performed. Patients still had subxiphoid and lower abdominal pain. The gastroenterologist recommended the addition of Delixin to regulate gastrointestinal nerves, gabapentin, continuous intravenous drips of hyoscyamine, and lidocaine for pain relief. The patient was discharged as her overall condition had changed, and she had regained energy. A genetic test was recommended to ascertain the diagnosis.

## 7. Follow-up and outcomes

The patient was contacted and informed of the diagnosis. We advised her on the AIP management process, the treatment options, and how each is done. The patient was advised to visit the hospital for prophylactic heme arginine injections in case of an attack. With the management treatment she had received, the patient did not have any attacks for two weeks since she was discharged. Furthermore, up to date, the patient has not had any attack.

## 8. Discussion

Porphyria is an inherited heme bioanabolic disorder caused by changes in specific enzymes in the heme biosynthesis pathway. It is a disease that can suddenly erupt and be life-threatening. ADP, AIP, HCP, and VP are the four types of multi-neurologic disorders in porphyria, and diagnosis is often delayed due to nonspecific symptoms. AIP, HCP, and VP are autosomal dominant disorders, and ADP is autosomal recessive.^[11]^ Porphyria symptoms are diverse, and biochemical analysis is necessary to diagnose an acute onset and determine the type of porphyria.^[12]^ Diagnosis of porphyria rarely requires genetic testing, which can be misleading if no mutations or unclassified variants are found.

AIP is the most serious of the acute porphyrias. PBG.D activity is reduced in both acute and latent patients. The studies of the PBG.D locus on the chromosome^[11]^ have shown significant molecular heterogeneity, also observed in the parents of one of the described homozygous cases of AIP. This heterogeneity has led to several phenotypic subtypes of the enzyme at the protein level, and this enzyme’s structure has a high degree of interspecific conservatism. Most human mutations occur in exons 10, 12, 58, and 63, consistent with alterations in the enzyme’s pyrrolidine cofactor binding site. Conveniently, biochemistry and X-ray crystallography revealed that enzymes from E. coli, slender-eyed worms, rats, and humans had 12 conserved arginine residues in the crack between the 3 protein domains, which would meet the need to bind 6 PBG molecules to 2 carboxyl groups each. The role of these conserved arginine residues has been investigated by replacing these residues in E. coli enzymes with leucine residues.

In contrast, the kinetic parameters of these mutant enzymes and the formation of intermediate enzyme-substrate complexes have shown that several arginine residues are involved in binding to the carboxylic acid side chains of pyrrolidine cofactors and the growing oligopyrrole chains. Mutant proteins synthesized by site-specific mutations exhibit a range of defects, including failure to assemble pyrrolidine cofactors and inability to initiate and propagate tetramerization reactions.^[13]^ Prepubertal symptoms of AIP are infrequent and more common in women than men. Symptoms of AIP occur due to visceral, peripheral, autonomic, and central nervous system effects, which usually occur intermittently and are sometimes life-threatening. The most commonly present are episodes of severe abdominal pain without peritoneal symptoms, often accompanied by nausea, vomiting, tachycardia, hypertension, anxiety, and agitation. Central nervous system manifestations may progress to confusion, hallucinations, and seizures. Typically, AIP does not affect the skin; however, concurrent advanced kidney disease can limit excretion and raise plasma porphyrin levels, resulting in blistering lesions on sun-exposed skin. Most of the synthesis of heme in the liver is used as a cofactor for a large number of CYPs, which are used for the detoxification of most drug metabolites. Thus, many drugs and hormones are inducers of ALA because they are inducers of Cytochrome P450 enzymes (CYPs), increasing the need for heme synthesis.^[14]^ Drugs are one of the most critical factors contributing to the acute onset of acute porphyria. Barbiturates and sulfonamide antibiotics are the most commonly used. Rifampicin or metoclopramide can also cure acute attacks. Drugs activating these patients’ cytochrome P450 enzyme system should be administered cautiously.^[15]^

Most patients with acute exacerbations require hospital admission, and about 1% of porphyria exacerbations can be fatal. For severe pain, opioids are safe to use, and other drugs like Pethidine and morphine can also be administered. Convulsions may occur during an acute attack caused by hyponatremia, so plasma osmolality and electrolyte values should always be checked. The treatment is fluid restriction, and the convulsions usually disappear as the seizures subside. Epilepsy may be a rare symptom of AIP, and therapy should be directed at the underlying disease. Oral and intravenous glucose and heme arginine salts are the mainstays of treatment, and gabapentin successfully controls seizures. A small proportion of patients (mainly women) have recurrent episodes without significant precipitation, and these patients may need to take prophylactic heme arginine regularly. In this setting, chemical menopause induction with luteinizing hormone-releasing hormone agonists has been successfully used.^[16]^ The mainstay of treatment is to prevent acute attacks as much as possible and avoid skin damage. Approximately 75% of acute attacks have identifiable triggers such as porphyrins, infections, malignancy, alcohol, tobacco, illicit drug use, fasting, pre-menstruation, stress like anemia, strenuous exercise, or travel. All these should be avoided. Patients with severe recurrent episodes requiring hospitalization may require regular weekly or nightly injections of prophylactic heme arginine. Erythropoietin is helpful in some patients, while liver transplantation is effective in severe case patients with near-persistent episodes. The only specific form of treatment is heme, which is thought to replenish the intracellular heme pool, inhibiting ALA synthase. However, heme instability may lead to renal failure, dose-related coagulopathy, and thrombophlebitis. Arginine heme has fewer side effects, is more stable than heme, and can be taken during an acute attack.^[17]^ Patients with recurrent episodes more than three times a year usually receive prophylactic administration of heme, which is intermittent therapy. However, frequent heme transfusions often result in venous occlusion because heme degradation products bind to endothelial cells, platelets, and clotting factors, and taking albumin-bound heme can reduce side effects.

In some cases, the frequency of clinical manifestations requiring intravenous heme may require up to weekly doses. High-dose intravenous heme (250 mg/d) can lead to heme oxygenase^[1]^ overexpression, leading to heme degradation and loss of ALAS1 feedback inhibition, as demonstrated in PBGD-deficient mice. This phenomenon was confirmed in PBGD-deficient mice. Recently, nano-specific PBGD RNAs have been shown to target liver cells, restore deficient enzyme activity, and protect mice from induced porphyria attacks. Notably, a novel drug (Givosiran) targeting the underlying pathology of AIP is expected to have high efficacy and tolerable side effects. Givosiran is a small interfering RNA that neutralizes excess ALAS1 mRNA in hepatocytes, and the small interfering RNA binds to the trimer N-acetylgalactosamine. After subcutaneous injection, Givosiran is guided to hepatocytes carrying N-acetylgalactosamine and binding to sialic acid-free glycoprotein receptors, which are subsequently endocytosed and small interfering RNAs split out of the conjugates, effectively reducing levels of ALAS1 mRNA and protein.^[8]^ Certain porphyrias have their unique treatments: for late-onset cutaneous porphyria, chloroquine treatment and iron removal by intravenous drainage are effective measures for the treatment of cutaneous porphyria; chloroquine forms a water-soluble complex with uroporphyrin and heparin carboxyl porphyrin, thereby increasing its clearance from tissues and excretion into the Urine.^[18]^ The highest-risk patients are those with significantly elevated plasma protoporphyrins, possibly high-throughput patients with intrahepatic porphyrin entry into bile. This liver disease is characterized by the deposition of protoporphyrin crystals in liver parenchymal cells, and liver transplantation is often the only effective treatment.^[19]^ Of all hepatic porphyrias, liver transplantation is most commonly used for AIP. Bone marrow transplantation (BMT) may cure EPP in patients with EPP after LT therapy, thereby preventing recurrent disease in allogeneic transplantation, but the timing is unclear. The indications for bone marrow transplantation are complex because of the lack of predictors of liver disease development in patients with EPP.^[20]^

Acute porphyria generally has a good prognosis if detected early and treated aggressively. In some patients, residual defects such as foot/wrist drooping or atrophy of the inherent muscles of the hand may occur. Patients with acute porphyria are at increased risk of developing cirrhosis and hepatocellular carcinoma, especially AIP. Therefore, all patients with acute porphyria over the age of 50 years should undergo lifelong screening for liver cancer, liver ultrasound, and serum alpha-fetoprotein measurement every 6 to 12 months.^[19]^

## 9. Conclusion

In summary, the diagnosis of acute intermittent porphyria is indeed complex because of the diverse clinical symptoms, which are similar to other diseases, and secondly due to the lack of specific auxiliary tests. The delay in diagnosis may result in severe damages or fatal cases. However, the presented case underscores the diagnostic complexity and treatment nuances of acute intermittent porphyria (AIP), emphasizing the importance of early recognition and a multidisciplinary approach. Identification of clinical manifestation symptoms is a guide for the diagnosis of AIP. According to the case report, patients with severe abdominal pain and neuropsychiatric symptoms should undergo urine testing for porphyrin, a diagnostic indicator of AIP. This case adds to the literature on early AIP identification to reduce its high mortality rate using the presented symptoms. It reminds clinical practitioners to consider the stated symptoms and steps taken to shorten the period before the diagnosis is made and proper treatment administered. Further research is warranted to enhance our understanding of AIP’s diverse manifestations and treatment outcomes, which could be unique to other patients.

## Author contributions

**Conceptualization:** Zhen Wang, Ruile Shen.

**Formal analysis:** Zhen Wang, Liniu Cheng.

**Supervision:** Ruile Shen.

**Writing – original draft:** Zhen Wang, Han Jiang.

**Writing – review & editing:** Zhen Wang, Xianyang Liang.
